# Editorial: Current Challenges in Cardiovascular Molecular Diagnostics

**DOI:** 10.3389/fcvm.2017.00054

**Published:** 2017-09-01

**Authors:** Valeria Novelli, Matteo Vatta

**Affiliations:** ^1^Institute of Genomic Medicine, Fondazione Policlinico Universitario Agostino Gemelli, Rome, Italy; ^2^Centro Studi “Benito Stirpe” per la prevenzione della morte improvvisa nel giovane atleta, Rome, Italy; ^3^Department of Medical and Molecular Genetics, Indiana University School of Medicine, Indianapolis, IN, United States; ^4^Invitae, San Francisco, CA, United States

**Keywords:** sudden cardiac death, next-generation sequencing applications, cardiovascular molecular diagnostic, variant interpretation, cardiovascular diseases

**Editorial on the Research Topic**

**Current Challenges in Cardiovascular Molecular Diagnostics**

In the last 10 years, the development of massive parallel sequencing technology, commonly referred to as next-generation-sequencing (NGS) technologies, carried the genetic field in a new era, opening unexplored avenues in the research of inherited cardiovascular disease (1).

As any new technology, when based on solid experimental observations and on real innovation, primarily generates a lot of enthusiasm among researchers as well as among patients, especially when it reaches a broader audience through the media coverage. However, as for every technology, along with the promises, the technical limitations inevitably appear, prompting more questions and further development. The technical refinement and the need for more robust and reliable assay validation standards delayed the introduction of NGS in clinical practice and the development of consensus standard operating procedure for the incorporation of NGS in the laboratory guidelines as the new standard test for the molecular diagnosis of inherited cardiovascular disease.

Here, we provide a brief review of the potential new applications and current challenges associated with the widespread use of NGS and the strategies that still need to be implemented to consider NGS a critical and sustainable tool for the diagnosis of cardiovascular diseases, and the detection of all at-risk family members.

In this research topic, we tried to raise awareness about the complexity of the issues that cardiologists and genetic practitioners have face. In particular, here we discuss the challenges in cardiovascular molecular diagnostics by targeting four aspects spanning different clinical specialties and timeframes, from the diagnosis to the treatment of patients. The management of patients affected by a genetic cardiovascular disease has changed substantially over the last decade. As a result, it is of critical importance to developed common managing strategies among multi-disciplinary stakeholders, and being able to synthesize the large quantity of information generated by the healthcare procedures, including the genetic testing laboratory, in order to provide the best care options for the patients and their families (2).

## Patients Selection and Indication to Genetic Testing

In the last years, thanks to the development of the concept of precision genomic medicine, an unprecedented proliferation of genetics- and genomics-guided testing has been proposed.

Although a variety of testing guidelines have been indicated by the European and American Societies of Cardiology (AHA, ACC, ESC, and HRS), the “real world” scenario is more heterogeneous; the optimal classification of individuals for molecular testing in inherited cardiovascular disease remains difficult (3). Genetic testing should be undertaken, indeed, only if considerable suspicion for an underlying genetic cardiovascular disease is present and always proposed after a comprehensive clinical evaluation, including, but not limited to, a detailed family history, cardiovascular work-up, and assessment for multisystem syndromes.

As we are all learning from NGS, the clinical utility of genetic testing is highly dependent on the pretest probability of each disease; common pitfalls associated with the inappropriate use of genetic testing, namely, poor phenotyping and inappropriate genetic test selection are very often able to hamper the diagnostic yield and the risk of encountering false-positive results.

## NGS Approach to Adopt

Next-generation sequencing can be applied to panels of genes, to the exome, namely the targeting of all coding exons, or the whole genome in clinical settings exome sequencing (ES) and genome sequencing (GS) are mainly adopted for gene discovery and used as clinical testing only when a clinical diagnosis cannot unequivocally be established. Differently, gene panels represent a good compromise between testing just a few genes and obtaining information from the exome. This approach is usually employed when a clinical diagnosis has been reached and does not lend itself to the identification of novel genes (4).

In order to fulfill the diagnostic necessities and homogenize the diagnostic procedures for different cardiac conditions, the design of custom target sequencing panels requires an in-depth knowledge of the specific disease and accuracy in the selection of the genes to analyze, according to their level of evidence.

The numbers of genes included in each panel can differ between laboratories. Some laboratories apply the strategy to include only “major genes” for which substantial literature is reported. Other panels include a larger gene set that includes the aforementioned major genes and additional “minor genes,” for which evidence is still accruing.

According to these considerations, it is important to highlight that when planning the development of a targeted gene panel, the main challenge is to define its main application and the targeted phenotypes for which the test is conceived.

## Variant Interpretation

The assessment of the pathogenicity of genetic variants is of critical importance. The high variability of the human genome calls to exercise extreme caution to avoid the misinterpretation of the identified genetic variants. Especially important for clinical genetic laboratories have been the development of large databases of control individuals, aiming at mimicking the genetic behavior of variants in a general population. When a variant is identified in a patient, we can now analyze its frequency in the largest open source database, namely the Genome Aggregation Database, which comprises of the data from sequencing 123,136 exomes and 15,496 genomes (http://gnomad.broadinstitute.org) (5). In addition to the variant frequency, evidence such as family studies, functional analysis, and biocomputational assessments need to be considered.

Deciding how to categorize and weigh each type of evidence is really challenging, and it is, therefore, difficult to validate approaches to variant assessment, particularly for variants that have limited evidence, usually identified by GS or ES.

This issue actually needs the collective experience of experts in the community to begin to build commonly validated approach to variant classification. Starting from the collaboration of a group of experts ACMG and Association for Molecular Pathology in 2015 developed a framework for evidence evaluation. This framework is now in revision in order to be personalized according to the gene variation (6).

## Sustainability of Genetic Testing in the “Real World”

When evaluating a genetic testing strategy, it is important to take into account the costs of that strategy and to determine if the increases in effectiveness are worth the additional costs that broader testing strategies incur. Genetic testing, although generally accepted by the medical community as an increasingly fundamental tool for patients’ management, remains a relatively expensive test for which, identifying who should bear the economic burden, remains often challenging. In particular, the heterogeneity of political and socio-economical systems make genetic testing a very different experience for patients and their family in various countries across the globe. Too often, the economic burden of genetic testing is loaded onto the pockets of the patients and their families, while apart from the few countries with a national program to fund genetic testing as every other clinical test, other systems rely on third parties for the management of medical expenses (7). However, contrary to what occurred with genetic testing for cancer, the equal acknowledgment of the service provided by genetic laboratories to thousands of cardiovascular patients has yet to be achieved.

## Remark Conclusive

The last few decades have brought much technological advances, which have forever changed the landscape of clinical genetic testing. Despite the natural enthusiasm for those changes, much remains to be done for the optimal application of the massive parallel sequencing technology we commonly refer to as NGS. Although NGS represents a powerful testing tool, it can reach its full potential only if integrated with improved clinical diagnostics, refinement of the sequencing strategy, standardized variant interpretation, and economic sustainability, which requires genetic testing to be embraced as standard clinical practice by the healthcare community in its entirety.

## Author Contributions

VN and MV contributed extensively to the work presented in this Editorial.

## Conflict of Interest Statement

The authors declare that the research was conducted in the absence of any commercial or financial relationships that could be construed as a potential conflict of interest.
